# Triple Synchronous Primary Neoplasms of the Cervix, Endometrium, and Ovary: A Rare Case Report and Summary of All the English PubMed-Indexed Literature

**DOI:** 10.1155/2017/9705078

**Published:** 2017-08-23

**Authors:** Ahmed Abu-Zaid, Mohannad Alsabban, Mohammed Abuzaid, Osama Alomar, Hany Salem, Ismail A. Al-Badawi

**Affiliations:** ^1^College of Medicine, Alfaisal University, Riyadh, Saudi Arabia; ^2^College of Graduate Health Sciences, University of Tennessee Health Science Center, Memphis, TN, USA; ^3^Department of Obstetrics & Gynecology, King Faisal Specialist Hospital & Research Centre, Riyadh, Saudi Arabia; ^4^Department of Obstetrics & Gynecology, King Fahad Medical City, Riyadh, Saudi Arabia

## Abstract

The incidence rate of triple or more synchronous primary neoplasms of the female genital system is exceedingly uncommon. To the best of our knowledge, only 13 such cases have been reported in the PubMed-indexed English literature. Herein, we report a single case of triple synchronous primary neoplasms of the cervix, endometrium, and left ovary with three distinct histological patterns that were not reported previously. Moreover, we briefly present a summary table of all the English PubMed-indexed cases of triple or more synchronous primary neoplasms of the female genital system (*n* = 13).

## 1. Introduction

Synchronous primary neoplasms are defined when two or more neoplasms take place concurrently in the same patient. These neoplasms should be histologically discrete and separated from each other by means of healthy tissues, such as basal lamina or stroma [1]. Double gynecological neoplasms are occasionally observed, and the most commonly reported combination is endometrial-ovarian neoplasms [2, 3]. The incidence of triple or more synchronous primary neoplasms of the female genital system is exceedingly uncommon. To the best of our knowledge, only 13 such cases have been reported in the PubMed-indexed English literature [1, 2, 4–14]. Herein, we report a single case of triple synchronous primary neoplasms of the cervix, endometrium, and right ovary with three distinct histological patterns that were not reported previously. Moreover, we briefly present a summary table of all English PubMed-indexed existing cases of triple or more synchronous primary neoplasms of the female genital system (*n* = 13).

## 2. Case Report

A 55-year-old multiparous woman was referred to our hospital as a case of pelvic/abdominal mass for 2 months. The mass was associated with progressive abdominal distention, left lower abdominal pain, and occasional vaginal bleeding. Her past medical and surgical histories were unremarkable. Upon pelvic examination, a cystic, mobile, nontender pelvic mass was palpated up to the umbilicus. There were no abnormal cervical growths. Laboratory findings showed a slightly elevated CA-125 level of 36.6 U/mL (normal range: 0–35 U/mL).

**Figure 1:**
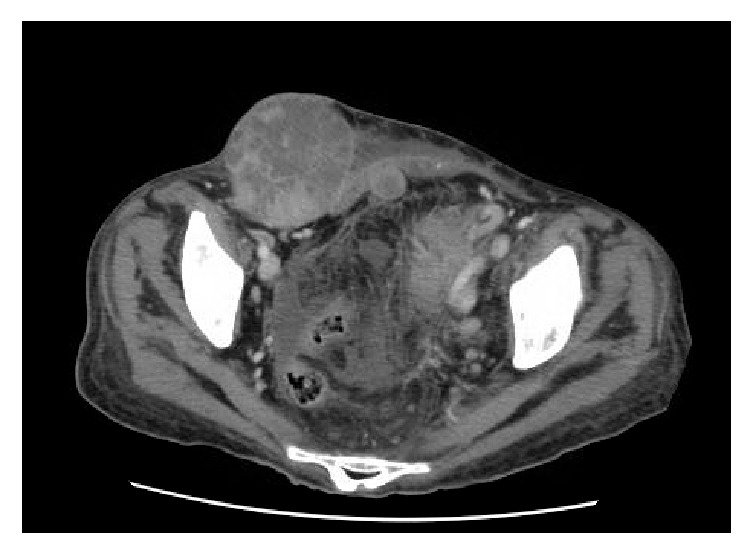
Axial CT scan showing heterogeneous 14 × 12 cm pelvic-abdominal mass between uterus and rectum, most likely originating from the left ovary and inseparable from the posterior uterus.

Two imaging studies were conducted: ultrasound (US) and computed tomography (CT) scan. US showed a 15 cm pelvic mass at the left adnexal site. The mass was cystic and contained solid areas. CT scan showed a heterogeneous 14 × 12 cm pelvic/abdominal mass with solid components, along with multiple prominent left pelvic and para-aortic lymph nodes that were highly suspicious for metastasis. The mass was seen between uterus and rectum. The mass was most likely originating from the left ovary and inseparable from the posterior uterus (Figure 1). In view of an underlying neoplastic process, a surgical staging operation was planned.

The surgical staging operation consisted of total abdominal hysterectomy, bilateral salpingo-oophorectomy, infracolic omentectomy, sampling from the pelvic lymph nodes and para-aortic lymph nodes, and multiple biopsy specimens from various peritoneal sites. During the laparotomy, the pelvic mass was found to be most likely originating from the left ovary. The uterus was bulky and the right ovary was grossly normal. There were no ascites or other gross intra-abdominal lesions. All resected specimens were examined for histopathological analysis.

**Figure 2:**
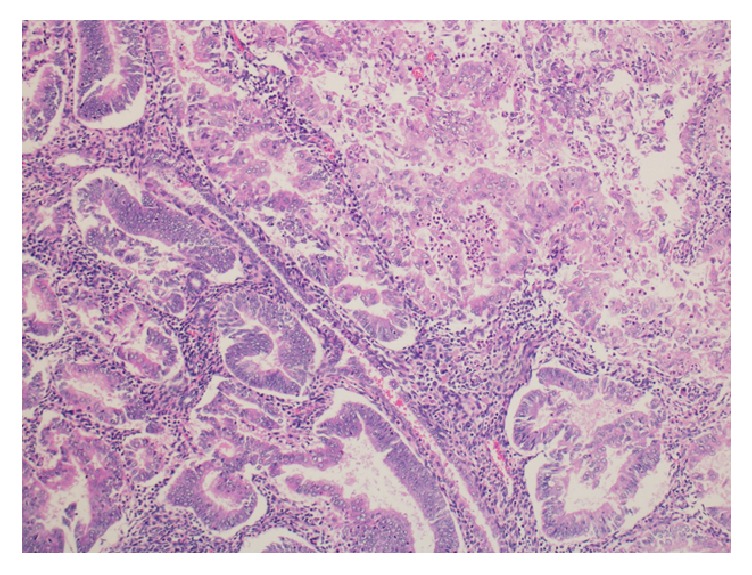
Histopathological examination showing grade-I endometrioid adenocarcinoma of the uterus (H&E stain).

**Figure 3:**
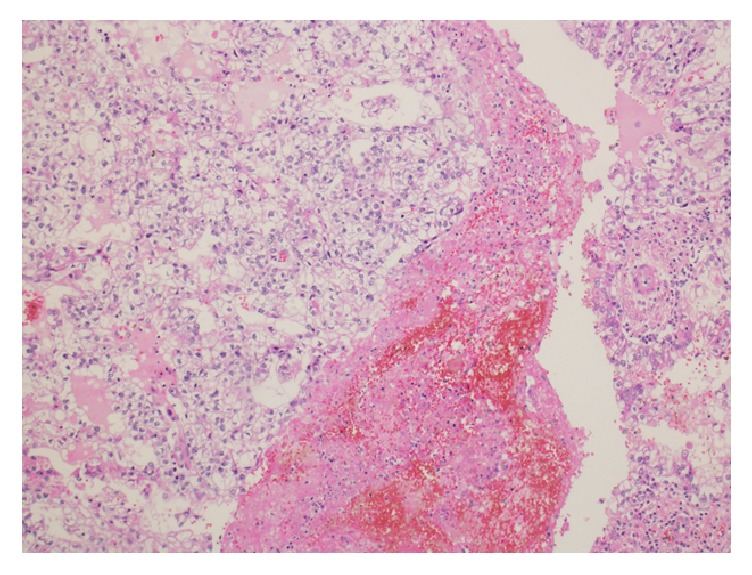
Histopathological examination showing clear-cell carcinoma of left ovary (H&E stain).

**Figure 4:**
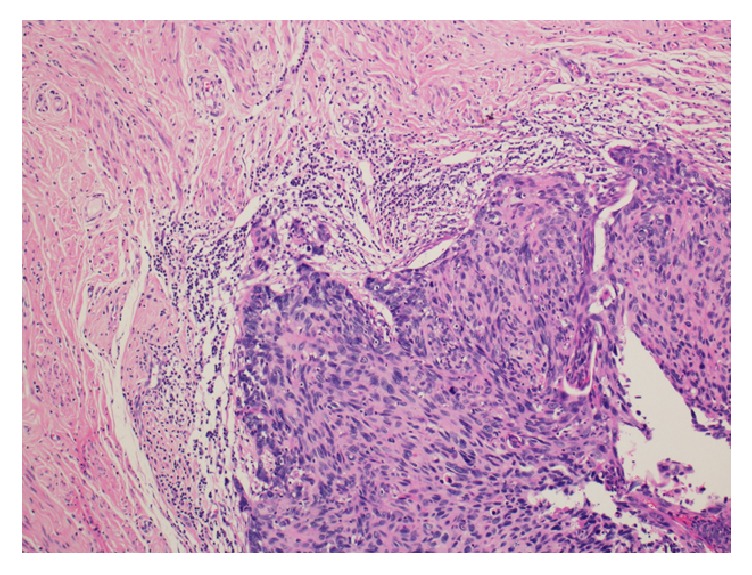
Histopathological examination showing poorly differentiated squamous cell carcinoma of the cervix (H&E stain).

Histopathological examination showed grade-I endometrioid adenocarcinoma of the uterus with no lymphovascular invasion and less than 50% invasion into the myometrium (Figure 2). Both ovaries were examined, and the microscopic appearance of the left ovary revealed clear-cell carcinoma with no lymphovascular invasion (Figure 3). The right ovary and bilateral fallopian tubes were normal. Microscopic examination of the cervix exhibited poorly differentiated squamous cell carcinoma (Figure 4). The omentum and bilateral pelvic and para-aortic lymph nodes were negative for metastasis.

Therefore, the final histopathological diagnosis was triple synchronous primary stage 1B1 poorly differentiated squamous cell carcinoma of the cervix (pTNM: T1B1 Nx M0), stage 1A grade-I endometrioid adenocarcinoma of the uterus (pTNM: T1A Nx M0), and stage 1A clear-cell carcinoma of the left ovary (pTNM: T1A Nx M0).

The case was discussed in the multidisciplinary tumor board meeting and the recommendation was to start adjuvant therapy. The adjuvant therapy consisted of chemotherapy and radiation therapy. The adjuvant chemotherapy was primarily intended for the high-risk ovarian cancer (clear-cell carcinoma) and included six cycles of paclitaxel 175 mg/m^2^ plus carboplatin (area under the curve [AUC] 6). The adjuvant radiation therapy was primarily intended for the high-risk cervical cancer (poorly differentiated squamous cell carcinoma) and consisted of external beam radiation therapy (EBRT) of 51 Gy delivered in 26 fractions.

The management plan was discussed with the patient; however, she refused the adjuvant treatment. Three months later, she presented to clinic with radiological evidence of recurrence, as follows: large local recurrence in the left iliac fossa, multiple metastatic masses in the abdominal and pelvic cavities, and multiple metastatic retroperitoneal and pelvic lymph nodes.

## 3. Discussion

The incidence rate of double synchronous primary gynecological neoplasms is relatively uncommon and ranges from 0.6% to 5.4% [2, 3, 15–17]. The incidence rate of triple or more synchronous primary neoplasms of the female genital system is exceedingly uncommon. To the best of our knowledge, only 13 such cases have been reported in the PubMed-indexed English literature [1, 2, 4–14] (Table 1). There was only one case of quintuple synchronous neoplasms [10] and two cases of quadruple synchronous neoplasms [8, 11]. On the other hand, there were only 10 cases of triple synchronous neoplasms. In our study, the distinctive combination of poorly differentiated squamous cell carcinoma of the cervix, grade-I endometrioid adenocarcinoma of the uterus and clear-cell carcinoma of the left ovary has never been previously reported.

The etiology of synchronous primary neoplasms of the female genital system remains poorly defined. It has been assumed that in genetically predisposed individuals, the Mullerian tissues with similar embryological origin may respond as a single structural entity when simultaneously exposed to carcinogenic, hormonal, therapeutic, or other triggering factors [2]. In our study, there were three different histological subtypes identified in the surgical specimens (squamous, endometrioid, and clear-cell). This unusual presentation raises thoughts about potential underlying epigenetic/bimolecular explanations, and this is an interesting arena for future research.

Several clinicopathological criteria have been suggested to assist clinicians and pathologists in distinguishing synchronous primary gynecological neoplasms from related metastatic foci. These criteria include either one major criterion or all the four minor criteria. The one major criterion is the existence of distinct histological types of the neoplasms. The four minor criteria include (a) neoplasms which are limited to primary locations, (b) absence of direct extension between neoplasms, (c) absence of lymphovascular neoplastic invasion, and (d) absence of distant metastasis [18, 19]. In our case, all the above-mentioned major and minor criteria were met, hence confirming the diagnosis of triple synchronous primary neoplasms of the female genital system.

It is critically crucial to differentiate between synchronous primary gynecological neoplasms and related metastatic diseases. This is because both management and prognosis vary substantially. Regarding prognosis, synchronous primary gynecological neoplasms are associated with better survival rates than metastatic or advanced primary ones [2, 3, 17]. This observation may be attributable to the younger age of presentation, earlier disease stage, and lower disease grade at the time of clinical diagnosis [17]. The prognosis of a triple neoplasm is largely determined by the neoplasm with the poorest prognosis [5].

There are no specific guidelines regarding the management of synchronous primary gynecological neoplasms. Proper management should be largely individualized taking into consideration several parameters, such as age of patient, disease type, disease stage, disease grade, and extent of the neoplastic invasion [10]. Management modalities include surgical debulking and/or adjuvant radiotherapy and/or adjuvant chemotherapy, as seen clinically appropriate. Patients with advanced stage, higher grade, and poor histological synchronous primary neoplasms should be treated with more aggressive management modalities.

Clear-cell carcinoma (CCC) of the ovary is regarded as a grade III and high-risk neoplasm, irrespective of the disease stage. Also, CCC of the ovary is recognized to be less sensitive to first-line platinum-based chemotherapy [20]. Previous evidence advocated that irinotecan was more effective against primary [21] and recurrent [22] ovarian CCC when compared to other cytotoxic regimens. However, a recent randomized phase III trial of paclitaxel plus carboplatin (PC) versus irinotecan plus cisplatin (CPT-P) in patients with ovarian CCC showed no superiority of CPT-P over the gold standard PC regimen [23].

Poorly differentiated squamous cell carcinoma of the cervix is regarded as a high-risk factor influencing the likelihood of recurrence. Hence, an adjuvant therapy may be recommended. The administration of concurrent cisplatin-based chemotherapy and radiation therapy (in the form of EBRT or brachytherapy) has been shown to substantially improve progression-free and overall survival for high-risk, early-stage patients with cervical cancer [24].

## 4. Conclusion

Although exceedingly uncommon, the likelihood of triple synchronous primary neoplasms should be considered when evaluating neoplasms of the female genital system. Careful pathological examination of the surgical specimens can substantially aid in recognizing such synchronous neoplasms.

## Figures and Tables

**Table 1:** A summary table of all existing cases of triple or more synchronous primary neoplasms of the female genital system (*n* = 13).

Ref	Author	Year	Age (yr)	Presentation	Site	Tumor histology	Tx	FU (mon)	Outcome
				Anorexia	Ovary	Papillary serous cystadenocarcinoma			
[14]	Matlock et al.	1982	77	Abdominal pain	Ovary	Mucinous cystadenocarcinoma	CT	4	DOD
				Weight loss	Uterus	papillary adenocarcinoma with psammoma bodies			

				NM	Ovary	Mucinous adenocarcinoma			
[2]	Ayhan et al.	1992	NM		Uterus	Endometrioid adenocarcinoma	NM	28	DOD
					Cervix	Carcinoma in situ			

				Vaginal bleeding	Ovary	Endometrioid adenocarcinoma			
[7]	Jobo et al.	1997	35		Uterus	Endometrioid adenocarcinoma	CT	25	NED
					Cervix	Carcinoma in situ			

				Vaginal bleeding	Ovary	Clear-cell carcinoma			
[1]	Ree et al.	2003	46		Ovary	Borderline mucinous cystadenoma	CT	17	NED
					Uterus	Endometrioid adenocarcinoma			

				Pelvic pain	Ovary	Mucinous adenocarcinoma			
[9]	Isin Dogan Ekici et al.	2006	56	Vaginal bleeding	Uterus	Endometrioid adenocarcinoma	CT	24	NED
					Uterus	Leiomyosarcoma			

				Menorrhagia	Ovary	Mucinous adenocarcinoma			
[11]	Phupong et al.	2007	50		Ovary	Low malignant potential	RT	3	DOD
					Uterus	Endometrioid adenocarcinoma			
					Cervix	Endocervical adenosquamous carcinoma			

				Spotting	Ovary	Brenner tumor			
[13]	Pekin et al.	2007	62	Vaginal bleeding	Ovary	Granulosa tumor	NM	NM	NM
					Cervix	Squamous cell carcinoma			

				Postmenopausal bleeding	Ovary	Mucinous adenocarcinoma			
[8]	Saglam et al.	2008	63		FT	Early papillary adenocarcinoma	CT	12	NED
				Abdominal distention	Uterus	Endometrioid adenocarcinoma			
					Cervix	Endocervical adenocarcinoma			

				Pelvic pain	Ovary	Papillary serous adenocarcinoma			
				Lower abdominal distention	FT	Microinvasive carcinoma in situ			
[10]	Atasever et al.	2009	50		FT	Microinvasive carcinoma in situ	CT	29	DOD
					Uterus	Intraepithelial adenocarcinoma			
					Cervix	Endocervical in situ carcinoma			

				Fatigue	Ovary	Mucinous, clear cell, and endometrioid carcinoma			
[6]	Hale et al.	2011	49	Ankle edema	Uterus	Endometrioid adenocarcinoma	NM	NM	NM
				Abdominal distention	Cervix	Endometrioid adenocarcinoma			

				Abdominal distention	Ovary	Leydig cell			
[12]	Zhang and Lerwill	2011	79	Abdominal pain	Uterus	Myxoid leiomyosarcoma	None	10	NED
				Dyspnea	Uterus	Mucinous adenocarcinoma			

				Metrorrhagia	Ovary	Serous adenocarcinoma			
[4]	Takatori et al.	2014	50		Uterus	Endometrioid adenocarcinoma	CT	18	NED
					Cervix	Endometrioid adenocarcinoma			

				Pelvic pain	Ovary	Mucinous adenocarcinoma			
[5]	Chiofalo et al.	2016	38	Vaginal bleeding	Uterus	Endometrioid adenocarcinoma	CT	18	NED
					Cervix	Mucinous adenocarcinoma			

				Abdominal distention	Ovary	Clear-cell carcinoma			
	Our case	2017	55	Abdominal pain	Uterus	Endometrioid adenocarcinoma	None	3	DP
				Vaginal bleeding	Cervix	Poorly differentiated squamous cell carcinoma			

Ref: reference; yr: years; Tx: therapy; mon: months; CT: chemotherapy; RT: radiotherapy; NM: not mentioned; DOD: died of disease; NED: no evidence of disease; DP: disease progression.
